# Shared and differential cortical functional abnormalities associated with inhibitory control in patients with schizophrenia and bipolar disorder

**DOI:** 10.1038/s41598-018-22929-y

**Published:** 2018-03-16

**Authors:** Noa Tsujii, Wakako Mikawa, Toru Adachi, Tomoyuki Hirose, Osamu Shirakawa

**Affiliations:** Department of Neuropsychiatry, Kindai University Faculty of Medicine, Osaka, Japan

## Abstract

Schizophrenia (SZ) and bipolar I disorder (BD-I) share genetic risk factors and cognitive impairments, but these conditions may exhibit differences in cortical functioning associated with inhibitory control. We measured hemodynamic responses during a stop-signal task using near-infrared spectroscopy (NIRS) in 20 patients with SZ, 21 patients with BD-I and 18 healthy controls (HCs). We used stop-signal reaction time (SSRT) to estimate behavioural inhibition. Compared with HCs, patients with either SZ or BD-I exhibited significantly reduced activation in the bilateral inferior, middle and superior frontal gyri. Furthermore, patients with BD-I showed inactivation of the right superior temporal gyri compared with patients with SZ or HCs. Patients with SZ or BD-I demonstrated significant negative correlations between SSRT and hemodynamic responses of the right inferior frontal gyrus. Moreover, patients with SZ exhibited correlations in the middle and superior frontal gyri. Our findings suggest that right inferior frontal abnormalities mediate behavioural inhibition impairments in individuals with SZ or BD-I. Differential patterns of orbitofrontal or superior temporal functional abnormalities may reflect important differences in psychopathological features between these disorders.

## Introduction

Although the traditional diagnostic classification in psychiatry cuts across the natural boundaries between schizophrenia (SZ) and bipolar I disorder (BD-I), recent epidemiological and molecular evidence supports shared genetic contributions between these disorders^1^. In addition, these psychiatric disorders share cognitive impairments that may be linked to structural and functional alterations in the frontotemporal cortex^2,3^. Deficits in inhibitory control, a major subcomponent of executive function, have been described in both SZ and BD-I. Inhibitory control is the ability to override, interrupt, or abort ongoing processes, especially when these processes are well engrained^3^. The stop-signal task can estimate the time required by an individual to withhold ongoing responses, as measured by stop-signal reaction time (SSRT)^4^. Compared with healthy controls (HCs), SSRTs are longer (i.e. suggesting impaired behavioural inhibition) in individuals with SZ^5^ or BD-I^6,7^.

Neuroimaging studies indicate that inhibitory processes involve the frontal cortex, particularly the right inferior frontal gyrus and presupplementary motor area^8–11^. Previous neuroimaging studies have found structural and functional abnormalities in these regions in individuals with SZ^12–14^ or BD-I^7,15,16^. In addition, functional magnetic resonance imaging (fMRI) studies have reported associations between behavioural inhibition and functional abnormalities of these brain regions in individuals with SZ^13,14^ or BD-I^17,18^. These neuroimaging studies suggest overlapping neural abnormalities in inhibitory processes between SZ or BD-I; however, it remains unclear to what extent these abnormalities have similar profiles or to what degree they differ between the two psychiatric disorders.

Near-infrared spectroscopy (NIRS) is a non-invasive optical technique for monitoring hemodynamic changes related to cortical neural activity by measuring relative changes in haemoglobin (Hb). Previous NIRS studies have reported functional abnormalities in the prefrontal cortex during a cognitive task in patients with SZ^19,20^ and BD-I (mainly individuals with bipolar II disorder)^21^. Recent studies have also reported that, compared with HCs, individuals with SZ show functional abnormalities of the bilateral prefrontal cortex during Go/No Go^22^ and stop-signal tasks^23^. Additionally, patients with bipolar II disorder show decreased oxygenated haemoglobin (oxy-Hb) changes in the bilateral orbitofrontal and left prefrontal cortices during the Iowa Gambling Task^24^. However, to our knowledge, no NIRS studies to date have directly compared functional abnormalities associated with inhibitory control in patients with SZ or BD-I relative to HCs.

In the current study, we investigated differences in cortical frontotemporal functional abnormalities associated with inhibitory control between patients with SZ or BD-I and HCs. We hypothesised that patients with SZ or BD-I possess shared–as well as different–cortical function patterns. These functional abnormalities appear to be directly associated with observed cognitive and clinical features, and these associations may characterise important differences in psychopathology between the two disorders.

## Results

### Demographic characteristics

The study participants consisted of patients with SZ (n = 20), BD-I (n = 21) and 18 HCs. Participants’ demographic data are shown in Table 1.

**Table 1:** Participant demographics and clinical characteristics.

	SZ		BD-I		HC		Statistics	*P* -value
	N	%	N	%	N	%		
Female, n (%)	11	55.0%	12	57.1%	10	55.6%	Χ^2^ = 0.02	0.990
Medication class, n (%)								
Antipsychotics	19	95.0%	9	42.9%			Χ^2^ = 12.9	0.000
Mood stabilisers	4	20.0%	12	57.1%			Χ^2^ = 5.9	0.015
Antidepressants	6	30.0%	8	38.1%			Χ^2^ = 0.3	0.585
	**Mean**	**SD**	**Mean**	**SD**	**Mean**	**SD**	**Statistics**	***P*** **-value**
Age, years	33.6	8.7	36.9	10.2	36.6	10.7	F(df = 2,56) = 0.70	0.505
Education, years	13.7	2.7	13.3	2.7	14.4	1.6	F(df = 2,56) = 1.1	0.347
Premorbid IQ^a^	103.3	9.3	105.4	7.9	106.3	9.9	F(df = 2,53) = 0.5	0.599
Handedness	93.0	11.7	77.1	40.6	87.2	17.4	F(df = 2,56) = 1.8	0.172
Duration of illness	9.2	4.8	10.4	7.3			t(df = 39) = 0.6	0.529
Antipsychotic dosage	498.1	446.1	126.2	183.7				
Antidepressant dosage	28.5	54.6	40.8	77.0				
GAF scores	49.6	16.3	52.8	16.2			t(df = 39) = 0.6	0.536
HAMD			10.3	7.3				0.000^b^
YMRS			2.5	2.6				0.568^b^
PANSS scores								
Total	15.4	5.1	9.0	4.2			t(df = 39) = 4.3	0.000
Positive	18.6	4.1	9.4	3.3			t(df = 39) = 7.8	0.000
Negative	36.8	7.6	29.4	8.5			t(df = 39) = 2.9	0.006
General	70.8	15.2	47.8	14.1			t(df = 39) = 5.0	0.000
Task performance								
Stop-signal delay	400.3	152.2	433.4	148.7	437.4	147.4	F(df = 2,56) = 0.36	0.70
Stop-signal reaction time	366.7	53.7	335.8	83.0	302.5	54.0	F(df = 2,56) = 4.5	0.015^c^
Percentage of miss trials	2.7	4.0	3.7	9.6	0.9	1.5	F(df = 2,56) = 0.99	0.38

SZ, schizophrenia; BD-I, bipolar I disorder; GAF, Global Assessment of Functioning scale; HC, healthy control; PANSS; Positive and Negative Syndrome Scale.

^a^Data were missing for two patients with SZ and one with BD-I.

^b^Analysed using Mann–Whitney U test.

^c^Patients with SZ demonstrated longer SSRTs than HCs (P = 0.011); no significant differences were observed between patients with BD-I and HCs (P = 0.26) or between the SZ and BD-I groups (P = 0.30).

### Task performance

SSRTs differed significantly across the SZ, BD-I and HC groups (P = 0.015; Table 1). Patients with SZ demonstrated longer SSRTs than HCs (P = 0.011). No significant differences were observed between with patients BD-I and HCs (P = 0.26) or between patients with SZ and BD-I (P = 0.30).

### Cognitive activation within groups

**Figure 1:**
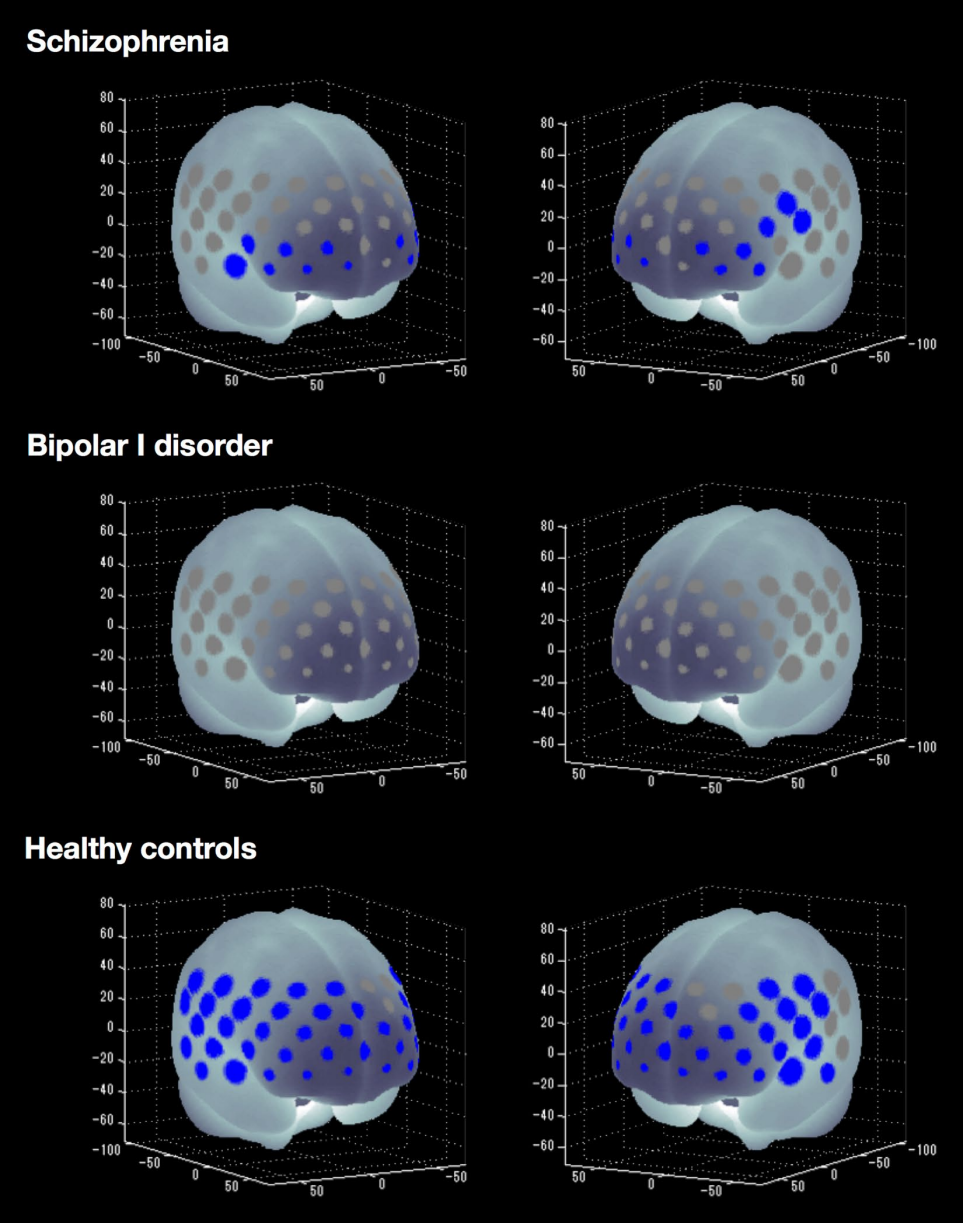
P-value significance map of cognitive activation among patients with SZ or BD-I and HCs (false discovery rate [FDR]-corrected *P* < 0.05, corrected with 52 channels). Blue-coloured circles represent significantly increased mean oxy-Hb changes from pre-task baseline to the inhibitory control task period in schizophrenia, bipolar I disorder and healthy controls. Channels that showed no significant correlations are highlighted in grey.

HCs showed significantly increased mean oxy-Hb levels between the pre-task baseline and the inhibitory control task period in 45 of 52 channels (‘ch’) (ch1–5, ch8–9, ch11–16, ch18–20, ch22–30, ch32–41 and ch43–52; t = −8.27 to −2.28; maximum false discovery rate (FDR)-corrected P < 0.05, corrected for 52 channels). Thus, widespread frontotemporal cortical activation related to oxy-Hb was induced by the inhibitory control task in HCs. Patients with SZ showed significantly increased mean oxy-Hb levels in 14 of 52 channels (ch19, ch29–30, ch34–36, ch38–39, ch44–47 and ch49–50; t = −4.22 to −2.76; FDR-corrected P < 0.05, corrected for 52 channels). By contrast, patients with BD-I did not show significant changes in oxy-Hb levels in any channel (FDR-corrected P < 0.05, corrected for 52 channels). Figure 1 summarises the cognitive activation results for all three groups.

### Regional differences in task-related oxy-Hb changes among groups

**Figure 2:**
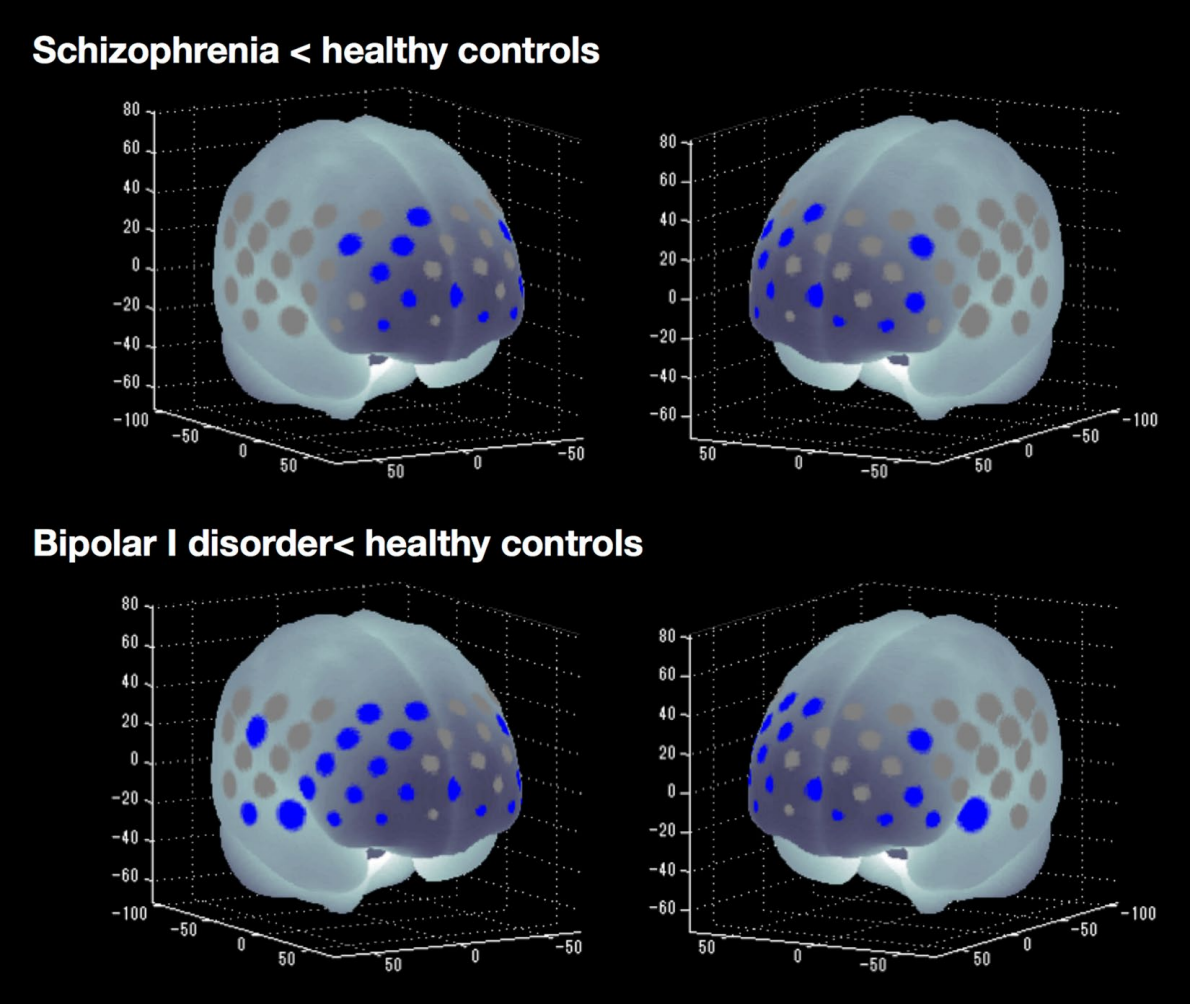
P-value significance map of post hoc analysis of task-related oxy-Hb changes in patients with SZ or BD-I compared with HCs (false discovery rate [FDR]-corrected *P* < 0.05, corrected with 52 channels). Blue-coloured circles indicate significantly smaller task-related oxy-Hb changes in the SZ or BD-I groups compared with HCs at the channels indicated. Channels that showed no significant correlations are highlighted in grey.

Task-related oxy-Hb changes differed significantly among the SZ, BD-I and HC groups in 21 channels (ch4–5, ch12, ch14–15, ch18, ch24–25, ch34–37, ch39, ch43–46, ch48–51; F = 4.23–11.18; FDR-corrected P < 0.05, corrected for 52 channels). Compared with HCs, the SZ group exhibited significantly smaller task-related oxy-Hb changes in 11 channels (ch5, ch14–15, ch18, ch25, ch36–37, ch39, ch46 and ch48–49; p = 0.009–0.049), whereas the BD group exhibited significantly smaller changes in 21 channels (ch4–5, ch12, ch14–15, ch18, ch24–25, ch34–37, ch39, ch43–46 and ch48–51; P = 0.000–0.044; Fig. 2). Thus, both patient groups produced significantly smaller task-related changes in oxy-Hb levels in the bilateral middle frontal, inferior frontal and superior frontal gyri compared with HCs (Table 2). Furthermore, patients with BD-I demonstrated significantly smaller task-related changes in oxy-Hb levels than patients with SZ or HCs in three channels (ch43–45; P = 0.015–0.036) located in the middle temporal and superior temporal gyri.

**Table 2:** Regional differences in task-related oxy-Hb changes among SZ, BD-I and HC groups.

Region	R/L	NIRS CH	Mean oxy-Hb changes		HC	F-value	Post hoc (*P*-value)		SZ vs. BD-I
			SZ	BD-I			SZ vs. HC	BD-I vs. HC	
**SZ < HC, BD-I < HC**									
Middle frontal gyrus	R	ch14	0.033 ± 0.070	0.025 ± 0.114	0.119 ± 0.092	5.72	0.019	0.008	0.954
		ch15	0.011 ± 0.063	0.011 ± 0.092	0.056 ± 0.102	5.20	0.019	0.018	1.000
		ch25	0.030 ± 0.106	0.031 ± 0.117	0.135 ± 0.082	6.07	0.009	0.009	0.999
		ch36	0.051 ± 0.069	0.033 ± 0.155	0.151 ± 0.138	4.62	0.049	0.016	0.890
	L	ch18	0.021 ± 0.055	0.034 ± 0.107	0.111 ± 0.116	4.62	0.017	0.044	0.906
		ch49	0.067 ± 0.109	0.015 ± 0135	0.186 ± 0.149	8.49	0.020	0.000	0.411
Inferior frontal gyrus	R	ch46	0.078 ± 0.106	0.014 ± 0.168	0.196 ± 0.140	7.57	0.042	0.001	0.326
	L	ch39	0.052 ± 0.074	0.030 ± 0.134	0.152 ± 0.107	6.52	0.020	0.003	0.791
Superior frontal gyrus	R	ch5	−0.006 ± 0.058	−0.026 ± 0.073	0.070 ± 0.125	5.95	0.033	0.005	0.760
		ch37	0.026 ± 0.130	0.036 ± 0.198	0.167 ± 0.115	4.71	0.020	0.034	0.980
	L	ch48	0.043 ± 0.096	0.019 ± 0.186	0.177 ± 0.130	6.10	0.019	0.005	0.857
**BD-I < HC**									
Middle frontal gyrus	R	ch4	0.014 ± 0.072	−0.015 ± 0.107	0.086 ± 0.123	4.75	0.092	0.011	0.657
Inferior frontal gyrus	R	ch24	0.054 ± 0.118	0.024 ± 0.132	0.133 ± 0.094	4.36	0.114	0.015	0.694
		ch34	0.108 ± 0.162	0.005 ± 0.142	0.162 ± 0.129	5.45	0.499	0.006	0.088
		ch35	0.094 ± 0.102	0.029 ± 0.147	0.167 ± 0.086	6.75	0.152	0.002	0.191
	L	ch50	0.072 ± 0.110	0.036 ± 0.150	0.154 ± 0.116	4.23	0.128	0.016	0.640
Postcentral gyrus	R	ch12	0.038 ± 0.112	−0.012 ± 0.123	0.118 ± 0.136	5.18	0.126	0.006	0.416
Superior temporal gyrus	L	ch51	0.099 ± 0.168	−0.026 ± 0.131	0.193 ± 0.192	7.94	0.195	0.001	0.062
**BD-I < SZ, BD-I < HC**									
Middle temporal gyrus	R	ch43	0.104 ± 0.159	−0.033 ± 0.163	0.157 ± 0.148	7.27	0.580	0.002	0.031
Superior temporal gyrus	R	ch44	0.128 ± 0.174	−0.050 ± 0.208	0.152 ± 0.164	6.39	0.920	0.005	0.015
Inferior frontal gyrus	R	ch45	0.120 ± 0.123	0.008 ± 0.158	0.220 ± 0.116	11.18	0.074	0.000	0.036

Abbreviations: SZ, schizophrenia; BD-I, bipolar I disorder; HC, healthy control.

In addition, analysis of covariance (ANCOVA) revealed that task-related oxy-Hb changes differed significantly among the SZ, BD-I and HC groups in 9 channels (ch5, ch34–35, ch43–46, ch49 and ch51; F = 5.41–11.59; FDR-corrected P < 0.05, corrected for 52 channels). The BD group exhibited significantly smaller task-related oxy-Hb changes in 9 channels in comparison to HCs (ch5, ch34–35, ch43–46, ch49 and ch51; P = 0.000–0.010) and in 3 channels in comparison to the SZ group (ch43–45; P = 0.004–0.021). However, ANCOVA did not show any significance between the SZ and HC groups after FDR correction.

**Figure 3:**
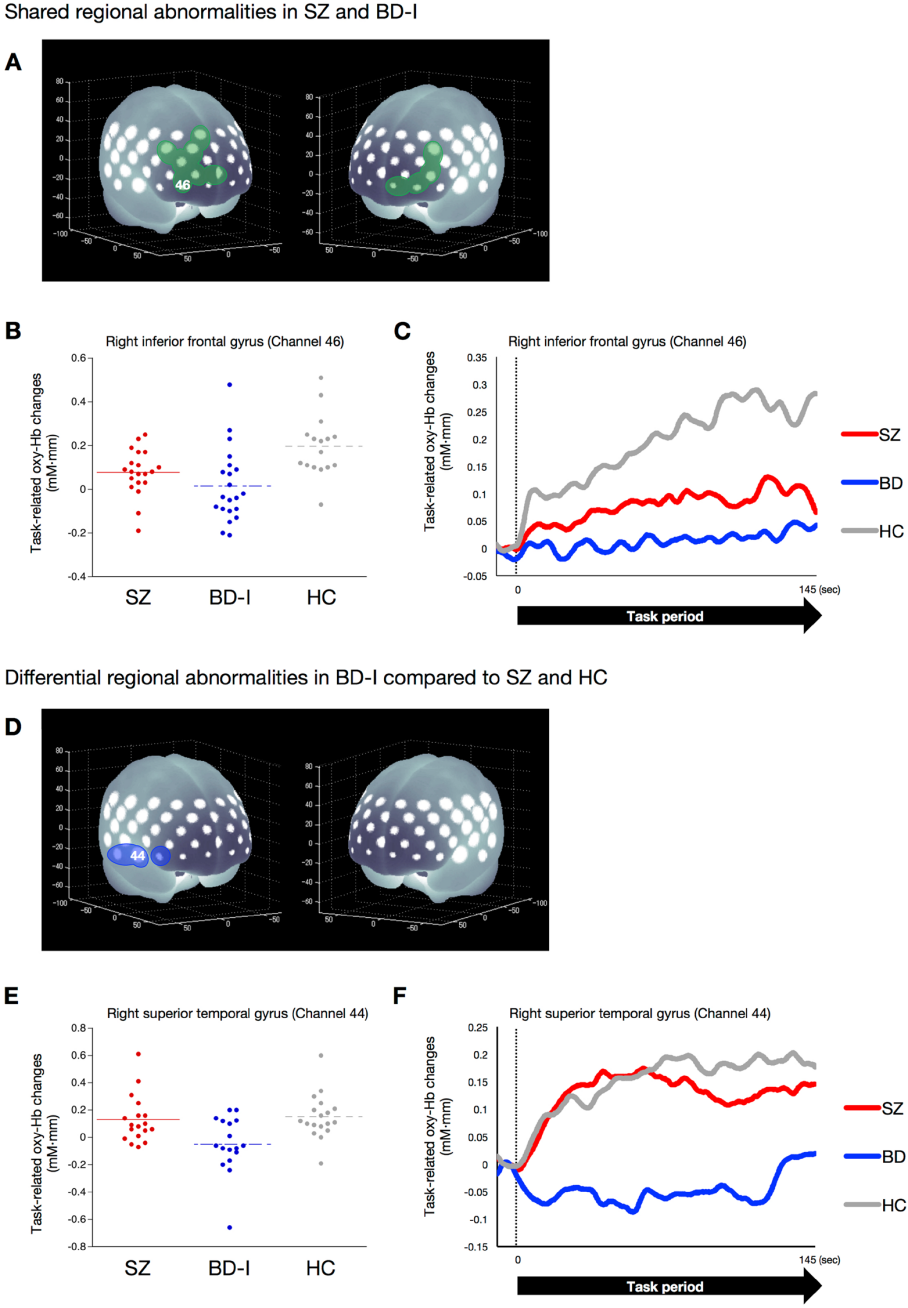
Shared or differential functional abnormalities in patients with SZ or BD-I compared with HCs. (**A**) Brain areas in green indicate regions with reduced hemodynamic responses, compared with HCs, overlapped in both patients with SZ and BD-I: bilateral inferior frontal, middle frontal and superior frontal (including orbitofrontal region) gyri. (**B**) Dot plots of task-related oxy-Hb changes in the right inferior frontal gyrus (channel 46) in the SZ, BD-I and HC groups. (**C**) Differential time course of task-related oxy-Hb signals in the SZ, BD-I and HC groups in the right inferior frontal gyrus (channel 46). In the HC group, the time course of changes in the task-related oxy-Hb signal showed a gradual increase; the SZ and BD-I groups did not exhibit this response. (**D**) Brain areas in blue indicate regions where reduced hemodynamic responses, compared with SZ and HC subjects, were observed in BD-I: right inferior frontal and superior temporal gyri. (**E**) Dot plots of task-related oxy-Hb changes in the superior temporal gyrus (channel 44) in the SZ, BD-I and HC groups. (**F**) Differential time course of task-related oxy-Hb signal in the SZ, BD-I and HC groups in the right superior temporal gyrus (channel 44). In the SZ and HC groups, the time course of changes in the task-related oxy-Hb signal showed a rapid increase; the BD-I group did not exhibit this response.

Figure 3 summarises the results of regional differences in task-related changes in oxy-Hb levels in patients with SZ or BD-I. In brief, reduced hemodynamic responses in the bilateral middle frontal, inferior frontal and superior frontal (including orbitofrontal region) gyri are a shared feature in SZ and BD-I. However, the reduction in the right superior temporal gyrus is specific to patients with BD-I.

### Correlation analysis

**Figure 4:**
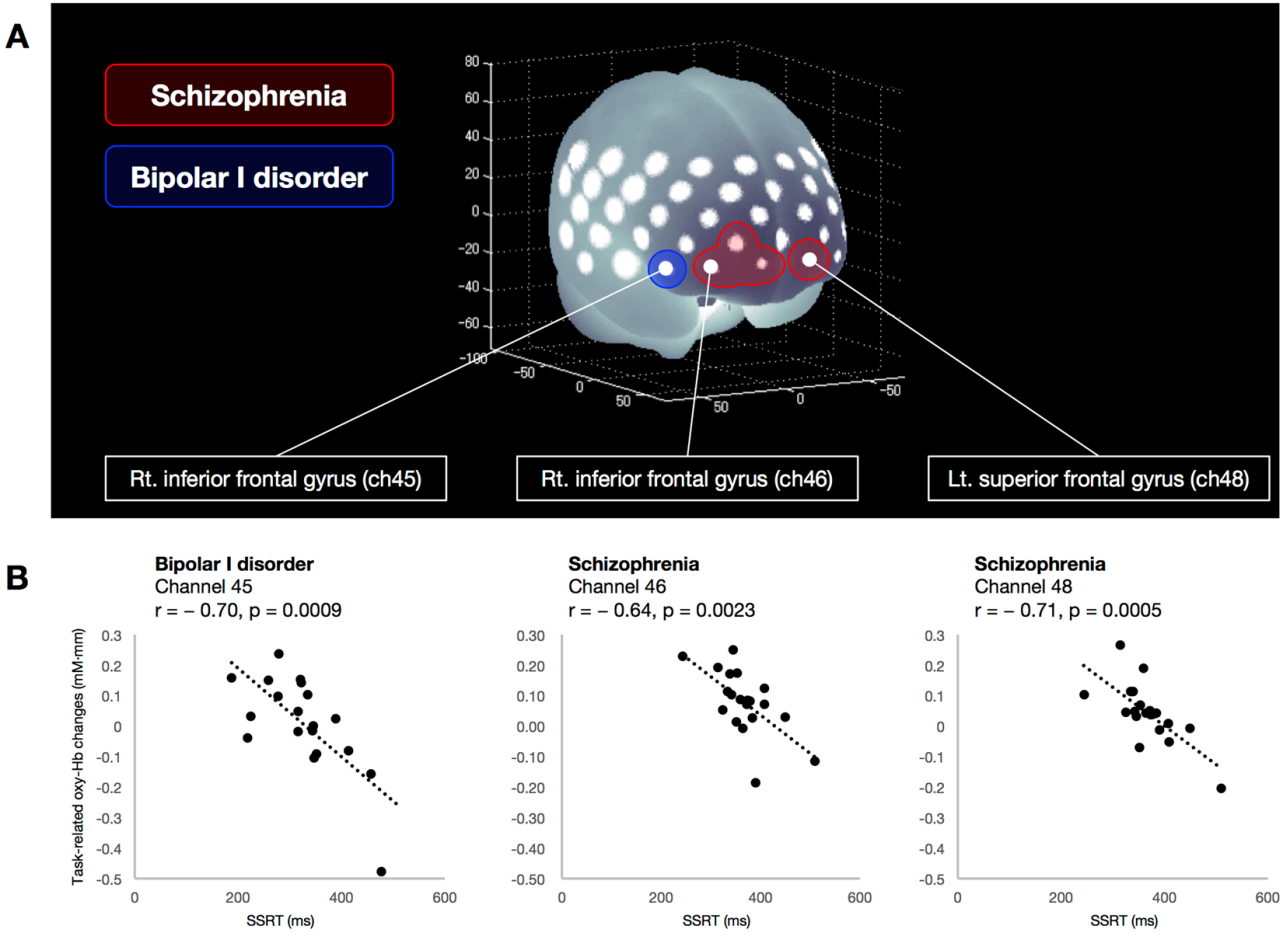
Shared or differential functional abnormalities associated with SSRT in patients with SZ or BD-I. (**A**) Brain areas in red indicate regions where task-related oxy-Hb changes negatively correlated with SSRT in the SZ group, and brain areas in blue indicate regions where task-related oxy-Hb changes negatively correlated with SSRT in the BD-I group. (**B**) Scatter plots showing the relationship between SSRT and task-related oxy-Hb changes in channels 45 and 46 (right inferior frontal gyrus) and 48 (right superior frontal gyrus).

Significant negative correlations between individual SSRT and task-related oxy-Hb changes were observed in the SZ group at four channels (ch36 and ch46–48; r = −0.71 to −0.64; FDR-corrected P < 0.05, corrected for 52 channels) located in the right middle frontal, inferior frontal and left superior frontal gyri, as well as in the BD-I group at one channel (ch45; r = −0.70; FDR-corrected P < 0.05, corrected for 52 channels) located in the right inferior frontal gyrus. There were no significant correlations between SSRT and task-related oxy-Hb changes in HCs. Figure 4 summarises the correlation analysis in patients with SZ or BD-I.

We observed significant negative correlations between the Global Assessment of Functioning (GAF) scale score and task-related oxy-Hb changes in the BD-I group at two channels (ch43–44; r = −0.72 to −0.68; FDR-corrected P < 0.05, corrected for 52 channels) located in the right middle and superior temporal gyri. No significant correlations were observed between task-related oxy-Hb changes and any other clinical variable in the SZ, BD-I, or HC groups, including antipsychotic or antidepressant dosage. No significant correlations were observed between task-related oxy-Hb changes and HAMD or YMRS scores in the BD-I group.

## Discussion

To our knowledge, this is the first NIRS study to directly compare regional cortical hemodynamic responses associated with inhibitory control among patients with SZ or BD-I and HCs. Patients with SZ or BD-I exhibited functional abnormalities associated with inhibitory control in the right inferior frontal gyrus. The differential abnormalities associated with behavioural inhibition were observed in the right middle and superior frontal gyri in patients with SZ and in the right superior temporal gyrus of patients with BD-I. Our findings suggest that differential patterns of frontotemporal functional abnormalities may reflect an important difference in psychopathology between schizophrenia and bipolar disorder.

Compared with HCs, patients with SZ or BD-I showed significantly reduced hemodynamic responses in the bilateral inferior frontal, middle frontal and superior frontal gyri. These results are consistent with previous SZ or BD-I neuroimaging studies that reported functional abnormalities in these regions^7,12–16^. The right inferior frontal gyrus is known to play a general role in attentional control, rapidly adapting inhibitory control responses to current salient stimuli^25–27^. Previous neuroimaging studies of HCs reported activation of the right inferior frontal and superior temporal gyri during a response inhibition task^28^. By contrast, studies have reported significant associations between poor inhibitory control and the volume of damage to grey matter in the right inferior frontal gyrus in patients with lesions of the right frontal lobe^29^ and reduced white matter fractional anisotropy in this region in patients with methamphetamine dependence^30^. Our results indicate that poor inhibitory control is associated with reduced hemodynamic responses in the right inferior frontal gyrus of patients with SZ or BD-I. These individuals have specific patterns of cerebral alterations in the right inferior frontal gyrus; this region may therefore be a neural substrate of impaired behavioural inhibition. Our findings suggest that right inferior frontal abnormalities mediate deficits in inhibitory control that are shared across patients with either SZ or BD-I.

In addition, the results of the current study suggest that patients with SZ exhibited deficits in inhibitory control associated with reduced hemodynamic responses in the right middle frontal and left superior frontal gyri, including the orbitofrontal region (channels 47–48). Previous studies on individuals with SZ indicated that structural or functional abnormalities in the orbitofrontal cortex were strongly associated with impulsive aggression or suicidal behavior^31–33^. Furthermore, alterations of orbitofrontal areas associated with SZ have been linked to positive and negative symptom profiles in previous structural neuroimaging studies^34,35^. Taken together, these studies suggest that functional abnormalities in the orbitofrontal region impair inhibition during impulsive or aggressive expression of symptoms in patients with SZ. Further, the alteration in this region may be related to the specific psychopathology of SZ. Our findings suggest that orbitofrontal functional abnormalities related to impaired behavioural inhibition are a neural substrate of the impulsive or aggressive clinical features associated with SZ.

By contrast, patients with BD-I demonstrated reduced hemodynamic responses in the right superior temporal gyrus, which was unique to this group. Moreover, patients with BD-I exhibited negative associations between SSRT and hemodynamic responses in this region. The superior temporal cortex (gyrus and sulcus) is part of a complex face-processing system involved in the perception of emotions^36^ and regulating responses to negative visual social stimuli^37^. Recent structural neuroimaging studies in patients with BD-I have found abnormally reduced thickness of the right superior temporal cortex^38^ or abnormally reduced fractional anisotropy in the right temporal lobe^39^. One investigation suggested that reduced activation in the superior temporal sulcus may indicate disturbances in affect-processing circuitry, leading to abnormalities in mood and social cognition^40^. These findings suggest that the superior temporal gyrus is an important neural substrate of emotion regulation and reward processing, and that alterations in this region may produce some of the cognitive symptoms and social impairments associated with BD-I. Moreover, functional abnormalities in this region may result in impaired emotional control inhibition in individuals with BD-I. This hypothesis is partly supported by the results of the current study, which showed an association between impaired global functioning and abnormal right temporal region hemodynamic responses in patients with BD-I. Our findings suggest that the functional abnormalities related to inhibitory control in the right superior temporal gyrus are characteristic of BD-I.

In addition, patients with SZ showed significantly longer SSRTs compared to patients with BD-I or HCs. This is consistent with previous evidence associating SZ with response inhibition deficits^2,5^. By contrast, no such difference in mean SSRT was observed patients with BD-I or HCs, consistent with the notion that neurocognitive functioning in individuals with BD-I is impaired relative to HCs but better than in patients with SZ^41,42^. An alternative explanation for this difference between SZ and BD-I groups is that the current mood state in patients with BD-I influenced SSRT, whereas the deficits persisted across all phases of BD-I^6,7,43^. A further explanation is that neuroimaging techniques, including the NIRS signal, are more sensitive than SSRT at detecting differences between the BD-I and HC groups, especially given small sample sizes. This observation has been reported in previous neuroimaging studies^17,18^. In the current study, we considered observed differences in hemodynamic responses among patients with SZ or BD-I and HCs to be activated by the stop-signal task.

There are several potential limitations to our study. First, our sample size was small, which may have limited the statistical power to produce reliable findings, i.e. ANCOVA did not show any significant differences between the SZ and HC groups after FDR correction. This could also explain why associations between SSRT and task-related oxy-Hb changes were not observed in HCs. In addition, the NIRS signal might be more sensitive, allowing greater detection of cortical functional abnormalities in patients with psychiatric diagnoses. This speculation is partly supported by previous NIRS studies that did not detect brain regions associated with task performances in HCs during Go/No Go^22^ and stop-signal tasks^23^. Further studies with larger sample sizes are needed to verify our findings. Second, we cannot exclude the possibility of medication effects on hemodynamic responses in patients with SZ and BD-I. However, it should be noted that the dosage of antidepressants or antipsychotics was not correlated with hemodynamic responses in any channels, suggesting a minimal confounding effect of medication. In addition, previous NIRS studies have reported no significant effect of psychotropic medications on neural activity in patients with SZ or BD^19–23^. Lastly, NIRS has lower spatial resolution to detect cortical activity from the scalp surface than other neuroimaging techniques such as fMRI. However, this limitation should be within an acceptable range because differences in hemodynamic responses in patients with SZ or BD-I and HCs were clearly observed in our study, as they have been in other NIRS studies^23,24,44^. In addition, we used a virtual spatial registration method to define the spatial information for each channel using data^45–47^. This method was used in most recent NIRS studies and may be useful for replicating our findings.

## Conclusion

This study demonstrated shared and differential patterns of hemodynamic responses in the frontotemporal cortex among patients with SZ or BD-I and HCs. Our findings suggest that differential patterns in frontotemporal functional abnormalities may reflect important differences in the psychopathological features of SZ or BD-I. NIRS is a non-invasive neuroimaging modality with easy applicability and high ecological validity. This makes NIRS particularly suitable for use with patients with psychiatric diagnoses who may be afraid of enclosed spaces or exhibit motor restlessness that interferes with motion-sensitive imaging methods^45^. Therefore, NIRS has the potential to be a powerful and specific diagnostic tool for use among individuals with SZ or BD-I.

## Methods

### Participants

The diagnosis of SZ or BD-I was made according to the Diagnostic and Statistical Manual of Mental Disorders, Fourth Edition^48^ criteria using the Mini International Neuropsychiatric Interview (MINI)^49^. HCs were also screened using the MINI and excluded if there was any history of psychiatric disorders or heritable neurological diseases among first- or second-order relatives. Exclusion criteria for the study groups were as follows: a history of head trauma with loss of consciousness for more than 5 min, current or previous neurological disease, current or previous endocrine disease, a history of electroconvulsive therapy and/or alcohol/substance abuse or addiction.

Psychiatric symptoms were assessed using the Positive and Negative Syndrome Scale (PANSS)^50^. Global functioning was assessed with GAF^48^. Depression severity was evaluated using the 17-item Hamilton depression rating scale administered using a structured interview guide^51^. Manic symptoms were assessed using the Young mania rating scale (YMRS)^52^. Premorbid IQ was estimated using the Japanese version of the National Adult Reading Test^53^. Handedness was evaluated according to the Edinburgh Inventory^54^. The chlorpromazine-equivalent dose of antipsychotics was calculated for each patient^55^.

After providing a complete description of the study, written informed consent was obtained from all participants. This study complied with the Declaration of Helsinki and was approved by the Ethics Committee of the Kindai University Faculty of Medicine. All data generated or analysed during this study are included in this article.

### NIRS methodology

**Figure 5:**
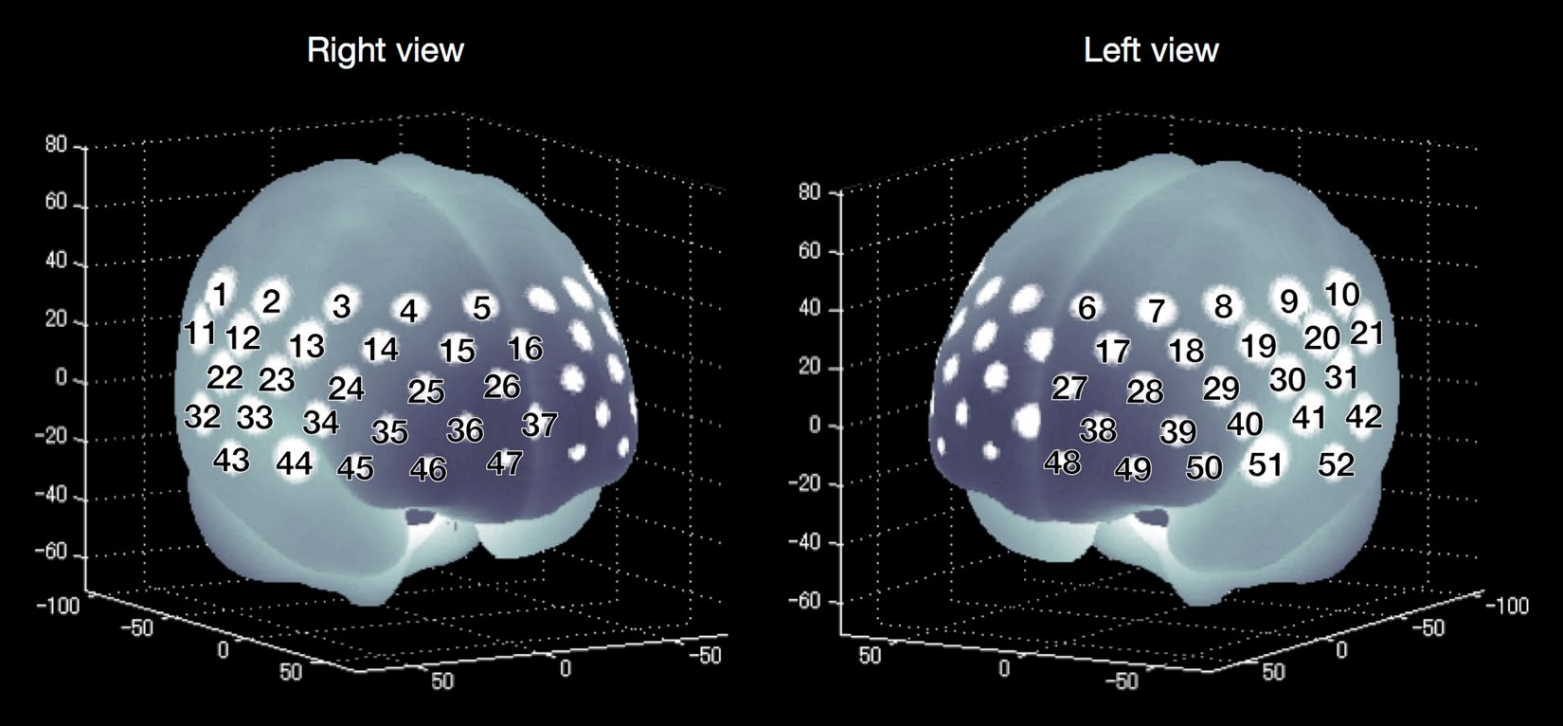
The locations of NIRS channels were probabilistically estimated and anatomically labelled in the standard brain space, in accordance with Tsuzuki *et al*.^54^.

We used a 52-channel NIRS device (ETG-4000 Optical Topography System; Hitachi Medical Co., Tokyo, Japan) to measure relative changes in oxy-Hb and deoxygenated haemoglobin (deoxy-Hb) at two wavelengths (694 and 830 nm) of near-infrared light (indicated as mM) based on the modified Beer–Lambert law^56^. The NIRS probes were fixed using 3 × 11 thermoplastic shells with 17 emitters and 16 detectors. The distance between each source and detector probe was 30 mm and the area analysed between the probes was defined as a ‘channel’. The probes of the NIRS device were placed on the frontotemporal region of each participant, with the lowermost probes located along the T3-Fp1-Fpz-Fp2-T4 line, in accordance with the International 10–20 Placement System used for electroencephalography. The NIRS device can measure Hb values bilaterally from the prefrontal and temporal surface regions at depths of 20–30 mm from the scalp, which correspond with the surface of the cerebral cortex. The spatial information for each channel was estimated using data from the Functional Brain Science Laboratory at Jichi Medical University, Japan^45,46^. According to the LONI Probabilistic Brain Atlas (LPBA40)^47^, NIRS channels can record functional hemodynamics within the bilateral frontal, temporal and parietal cortices. We anatomically labelled NIRS channels only after the LPBA40 region of highest probability was determined (Fig. 5).

We recorded relative mean changes in Hb concentrations from baseline in mM because NIRS cannot measure the absolute path length from the emitter to the detector. NIRS signals were acquired with a time resolution of 0.1 s. We set the moving average window to 5 s to remove high frequency noise such as heartbeat and small bodily movements. Channel records with low signal-to-noise ratios or motion artefacts were excluded, and there was no difference in the number of samples at each channel, including the channels that indicated significant differences among study groups.

In the present study, we focused on changes in oxy-Hb. Animal studies indicate that oxy-Hb is the most sensitive indicator of regional cerebral blood flow because the direction of change in deoxy-Hb is determined by the degree of change in venous blood oxygenation and volume^57^. Pre-task baseline was determined as the mean across the last 10 s of the 30 s pre-task segment. The obtained oxy-Hb data were averaged for each participant during each baseline and task period. The task-related oxy-Hb changes (task minus baseline) were used for statistical analysis.

### Stop-signal task

We used a variation on the classic stop-signal task^4^ (SST) to assess inhibitory control^58^. The original task design has been described in detail^58,59^. In brief, across the task period, participants viewed the shape of a ‘go’ stimulus (circle or square) that appeared on screen in rapid succession. Occasionally, an auditory stop signal was presented at a short, variable delay after the onset of the go stimulus.

### Procedure

**Figure 6:**
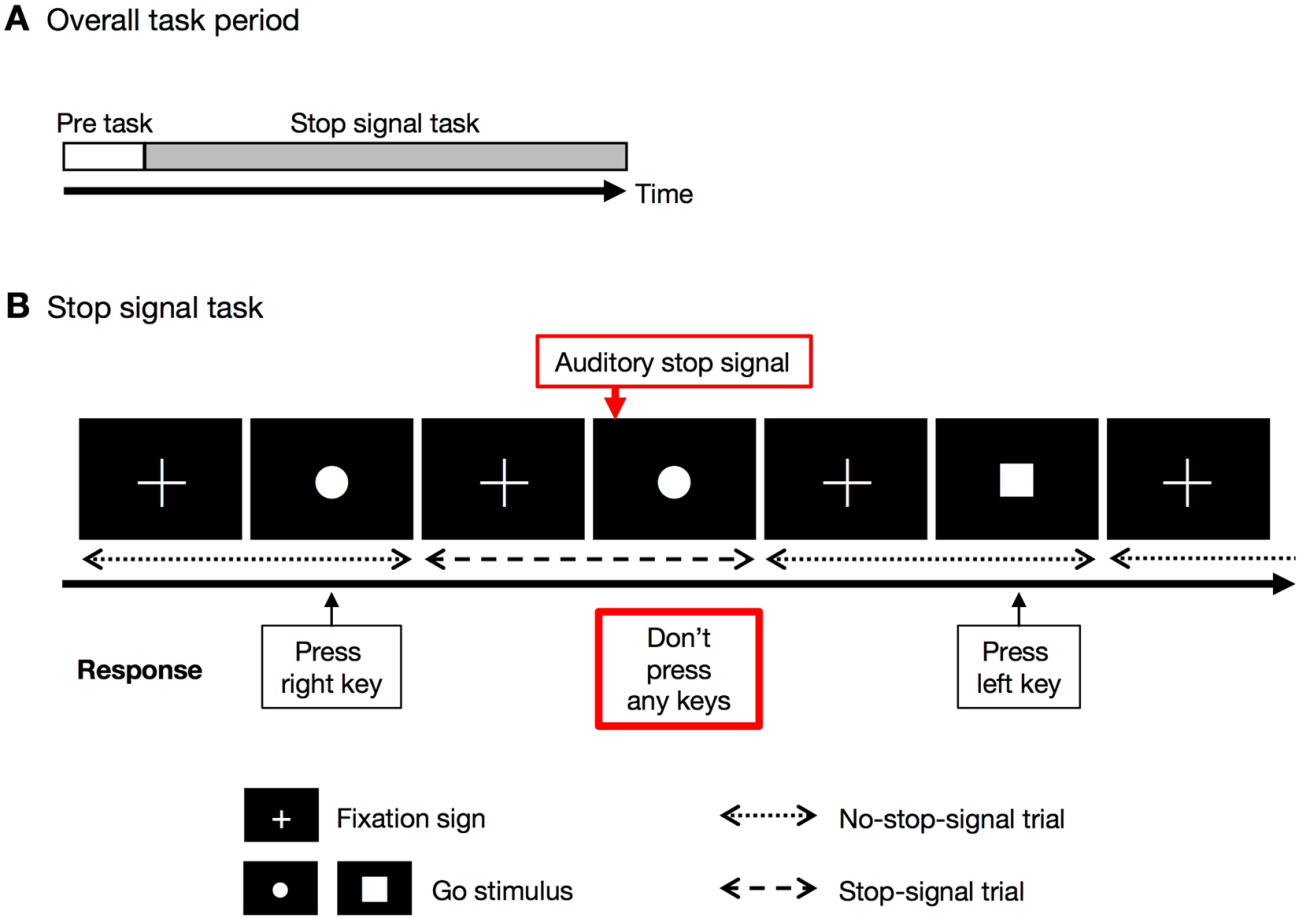
Task segments in the NIRS measurements and depiction of a trial in the stop-signal paradigm. (**A**) Overall task period. During the pre-task period, subjects were instructed to alternatively tap with their right and left index fingers every 1 s as accurately as they could. (**B**) During the stop-signal task, subjects were instructed to respond as quickly and accurately as possible to the go stimulus (‘●’, ‘■’) in no-stop-signal trials. In stop-signal trials, participants were instructed to try to withhold their response as required until the auditory stop signal occurred.

The cognitive activation task used in this study had a one-block design and included 1) a pre-task baseline period and 2) a stop-signal task period (Fig. 6A). During a 30 s pre-task period, participants were instructed to alternatively tap with their right and left index fingers every 1 s as accurately as they could, using the left and right buttons on a keyboard. The task consisted of a block of 64 trials; stop-signal trials (75% of trials) and non-stop-signal trials (25% of trials) were randomly presented. Each trial started with the presentation of a fixation sign on a computer monitor, which was replaced by the primary task stimulus after 250 ms. Stop-signal delay was initially set at 250 ms and continuously adjusted with the staircase tracking procedure (range of 250–1050 ms). When inhibition was successful, the stop-signal delay increased by 50 ms; when inhibition was unsuccessful, it decreased by 50 ms. Response registration continued during stop-signal presentation. In stop-signal trials, the go stimulus was followed by a stop signal (750 Hz, 75 ms) after a variable stop-signal delay. In both no-stop-signal trials and stop-signal trials, the go stimulus remained on screen until subjects responded or the maximal reaction time (RT; 1,250 ms) had elapsed. The inter-trial interval was 2,000 ms and independent of RT. Participants were instructed to respond as quickly and accurately as possible to the go stimulus in non-stop-signal trials. In stop-signal trials, participants were instructed to try to withhold their response as required until the auditory stop signal occurred (Fig. 6B).

### Statistical analysis

Demographic and clinical variables were compared among the study groups using the χ^2^ test, *t*-test and one-way ANOVA followed by Tukey’s *post hoc* test. Statistical significance was assumed at P < 0.05 (two-tailed).

To identify regional differences in frontotemporal hemodynamic activation associated with the inhibitory control task, we compared mean oxy-Hb changes during the pre-task (baseline) and task periods for every channel within individual subjects using the paired *t*-test. Task-related oxy-Hb changes were compared among the study groups using ANOVA followed by Tukey’s *post hoc* test. Because Tukey’s *post hoc* test focused on NIRS channels where the hemodynamic response was shown to be significant after FDR correction, no further correction for multiple comparisons was applied for the post hoc test and responses were considered significant at *P* < 0.05^23^. Complementary analyses were performed to identify between-group differences in activation associated with inhibitory control using ANCOVA with age, gender (dummy parameterised, male = 0, female = 1) and SSRT as covariates. Furthermore, to examine the relationships between task-related oxy-Hb changes and clinical variables, we calculated Pearson’s correlation coefficients. We set the value of q (i.e. FDR) to 0.05 such that the false-positive rate was not greater than 5% on average when processing oxy-Hb data obtained from multiple channels^60^.
